# *Primuladongchuanensis* (Primulaceae), a new species from northern Yunnan, China

**DOI:** 10.3897/phytokeys.130.35047

**Published:** 2019-08-29

**Authors:** Zhi-Kun Wu, Fu-Wei Zhao, Jia-Hui Chen, Yuan Huang

**Affiliations:** 1 Department of Pharmacy, Guizhou University of Traditional Chinese Medicine, Guiyang, 550025, China Guizhou University of Traditional Chinese Medicine Guiyang China; 2 Nanjing Institute of Environmental Sciences, Ministry of Ecology and Environment, Nanjing, 210042, China Nanjing Institute of Environmental Sciences Nanjing China; 3 Kunming Institute of Botany, Chinese Academy of Sciences, Kunming, 650204, China Kunming Institute of Botany, Chinese Academy of Sciences Kunming China; 4 Engineering Research Center of Sustainable Development and Utilization of Biomass Energy, School of Life Sciences, Yunnan Normal University, Kunming, 650001, China Yunnan Normal University Kunming China

**Keywords:** Primulaceae, *Primuladongchuanensis*, new species, Yunnan, China

## Abstract

*Primuladongchuanensis* Z.K.Wu & Yuan Huang, a new species of Primulaceae from Dongchuan of northern Yunnan, China, is described and illustrated. Both morphological and molecular evidence support *P.dongchuanensis* as a member of the sect. Proliferae. It is similar to *P.aurantiaca* W.W.Smith & Forrest, but is distinguished by having unique raceme inflorescences. Its distribution, phenology and conservation status are also provided.

## Introduction

*Primula* Linn. is one of the largest genera of Primulaceae, including about 500 species worldwide. Most *Primula* species are indigenous to the north temperate zone, with only a few outliers on some mountains of Africa (Ethiopia), tropical Asia (Java and Sumatra) and South America (Hu 1994, Hu and Kelso 1996, Mast et al. 2001). The modern center of diversity of *Primula* is in southwestern China, with ca 300 species of 24 sections, most of which occur in western Sichuan, eastern Xizang, and northwestern Yunnan (Hu 1994, Hu and Kelso 1996). Increased exploration across this region results to the discovery and description of new *Primula* species in the past 15 years (Hu and Geng 2003, Wu et al. 2013, Xu et al. 2017a, 2017b, Yang et al. 2017, Ju et al. 2018)

Section Proliferae Pax (10:217, 1889) of the genus *Primula* comprises more than 20 species, mainly distributed in Eastern Himalaya and Hengduan Mountain in China. Most species in this section are horticulturally important plants. Morphologically, sect. Proliferae shows the distinct character of several whorls of flowers in superimposed umbels and is recognized as a ‘natural’ group in this genus. Previous studies presumed that the sect. Proliferae may represent the most primitive group of *Primula* alive today, and take a central position with respect to subsequent evolution and geographical migration in the genus (Richards 1993, 2002). However, molecular phylogenetic evidence posited the opposite conclusion and indicated that the sect. Proliferae represents relatively advanced members of *Primula* that exist today (Mast et al. 2001, 2004, Yan et al. 2015).

During the field investigation in the Jiaozi Snow Mountain in Dongchuan of Yunnan, southwestern China in 2011, we found a peculiar population of *Primula* in its vegetative stage on a small patch of alpine meadow near the mountain top. We transplanted some living individuals to Lijiang Alpine Botanical Garden (at elevation of ca. 3200m), northwest of Yunnan and they regained their bloom in subsequent years. The plant has a short rootstock and robust fibrous roots, obovate-oblong to oblanceolate leaves forming a dense rosette and flowers showed great similarity to the species of sect. Proliferae, except the inflorescences with obsolete scapes at early anthesis, then elongating to forming raceme at late flowering. We presumed the unusual inflorescence springs from abnormal variations of plant response to a different climate zone and soil type after translocation. After the field investigations in the same locality in 2016 and in 2019, we confirmed that the inflorescences we observed from the translocated individuals are morphologically consistent with those of the wild population. Further molecular phylogenic analysis revealed it is an undescribed taxon of sect. Proliferae. We concluded that the species is new to science and describe it here.

## Materials and methods

Morphological descriptions and comparisons were based on living plants from the Lijiang alpine botanical garden and in the field, specimens from the herbarium of Kunming Institute of Botany, Chinese Academy of Sciences (KUN), and literatures (Chen and Hu 1990, Hu and Kelso 1996). All morphological characters of *P.dongchuanensis* and its morphological similar species *P.aurantiaca* were measured using a vernier caliper. The conservation status of *P.dongchuanensis* was assessed according to the IUCN Red list Categories and Criteria (IUCN 2017).

Genomic DNA was isolated from silica gel-dried leaves using a modified Cetyl Trimethyl Ammonium Bromide (CTAB) protocol (Doyle and Doyle 1987). The nrDNA and two chloroplast *matK* and *trnH-psbA* regions of *P.dongchuanensis* were amplified and sequenced using previously published universal primers (White 1990, Taberlet et al. 1991, Kress and Erickson 2007, Yang et al. 2012). Sequences of the relatives of *P.dongchuanensis* were downloaded from NCBI (https://www.ncbi.nlm.nih.gov/) (Appendix 1). Sequences for each region were aligned with CLUSTALX (Thompson et al. 1997) and then manually adjusted in BIOEDT 7.0 (Hall 1999). Maximum likelihood (ML) methods for phylogenetic estimation were conducted using IQ-TREE v. 1.6.10 under the GTR+G model (Nguyen et al. 2015). Clade supports were evaluated by 10000 bootstrap replicates of nonparametric approximate likelihood-ratio test (SH-alRT) and ultrafast bootstrap approximation approach (UFBoot) (Guindon et al. 2010; Hoang et al. 2018). Pairwise genetic distances among *P.dongchuanensis* and its closest relatives revealed by phylogenetic analyses were calculated using the Kimura 2-parameter method (Kimura 1980).

## Taxonomic treatment

### Primula dongchuanensis

Taxon classificationPlantaeEricalesPrimulaceae

Z.K.Wu & Yuan Huang sp. nov.

0B7E8E4305AF5F14A9B2FC3FE15C8680

urn:lsid:ipni.org:names:77201399-1

Figs 1 , 2 , 3A

#### Diagnosis.

The new species most resembles *P.aurantiaca*, sharing a similar flower color, leaf shape, efarinose and glabrous, and long calyx parted below the middle. But it can be distinguished by having much smaller statue, inflorescence raceme, scapes nearly obsolete at early anthesis and deep yellow flowers. The main morphological differences between *P.dongchuanensis* and *P.aurantiaca* are summarized in Table 1.

**Table 1.** Morphological and phenological comparisons between *Primuladongchuanensis* and *P.aurantiaca*.

Characters	*P.dongchuanensis*	*P.aurantiaca*
Leaf blade	3–6 × 2.0–3.5 cm	4–15 × 1.8–5.0 cm
Scape	scape nearly obsolete at early anthesis, elongating to 10 cm at late flowering	scape 4.5–15 cm at anthesis, elongating to 30 cm in fruit
Inflorescence	inflorescences 6–20-flowered arising from leaf rosette at early anthesis, elongating to 10 cm with 2–8 flowers forming solitary racemes at late flowering	umbels 2–4(–6), superimposed, 6–15-flowered
Pedicels	pedicel green, 1–3 cm long, glabrous	pedicel reddish, 0.3–1.0 cm long, glabrous
Bracts	1–2, linear	1, linear
Flower color	deep yellow	deep reddish orange
Flowering time	late April to early June	late May to early July

#### Type.

CHINA. Yunnan: Jiaozi Snow Mountain, Dongchuan district, ca. 3860 m, 102°55.75'E, 26°9.45'N, July 2016, *Z. K. Wu & Yuan Huang, ZKWu2016060* (holotype: KUN!; isotype: KUN!).

#### Description.

Perennial efarinose herb, glabrous, with a short root stock and 5–10 robust fibrous roots. Leaves forming a dense rosette, leaf blade obovate-oblong to oblanceolate, 3–6 × 2.0–3.5 cm, base attenuate, decurrent to petiole, margin erose-denticulate, apex rounded, petiole slightly differentiated to 1/3 as long as leaf blade; Scapes nearly obsolete with “compressed” 6–20-flowered inflorescences arising from leaf rosette at early anthesis, elongating up to 10 cm with 2–8 flowers forming solitary racemes at late flowering; bracts 1–2, linear, 1.0–1.8 cm long, glabrous. Pedicel 1–3 cm, glabrous. Flowers heterostylous. Calyx tubular-campanulate, 6–9 mm long, lobed to 1/2 of its length; lobes lanceolate, each with one prominent midvein, acuminate at apex. Corolla deep yellow; limb 1.2–1.8 cm wide; lobes oblong-obovate, emarginate. Pin flowers: corolla tube 0.8–1.2 cm long; stamens ca. 5 mm above base of corolla tube; style ca. 9 mm. Thrum flowers: corolla tube 0.9–1.4 cm long; stamens ca. 1.2 cm above base of corolla tube; style ca. 5 mm. Capsule subglobose, ca. 4 mm in diameter, ca. as long as calyx.

**Figure 1.**
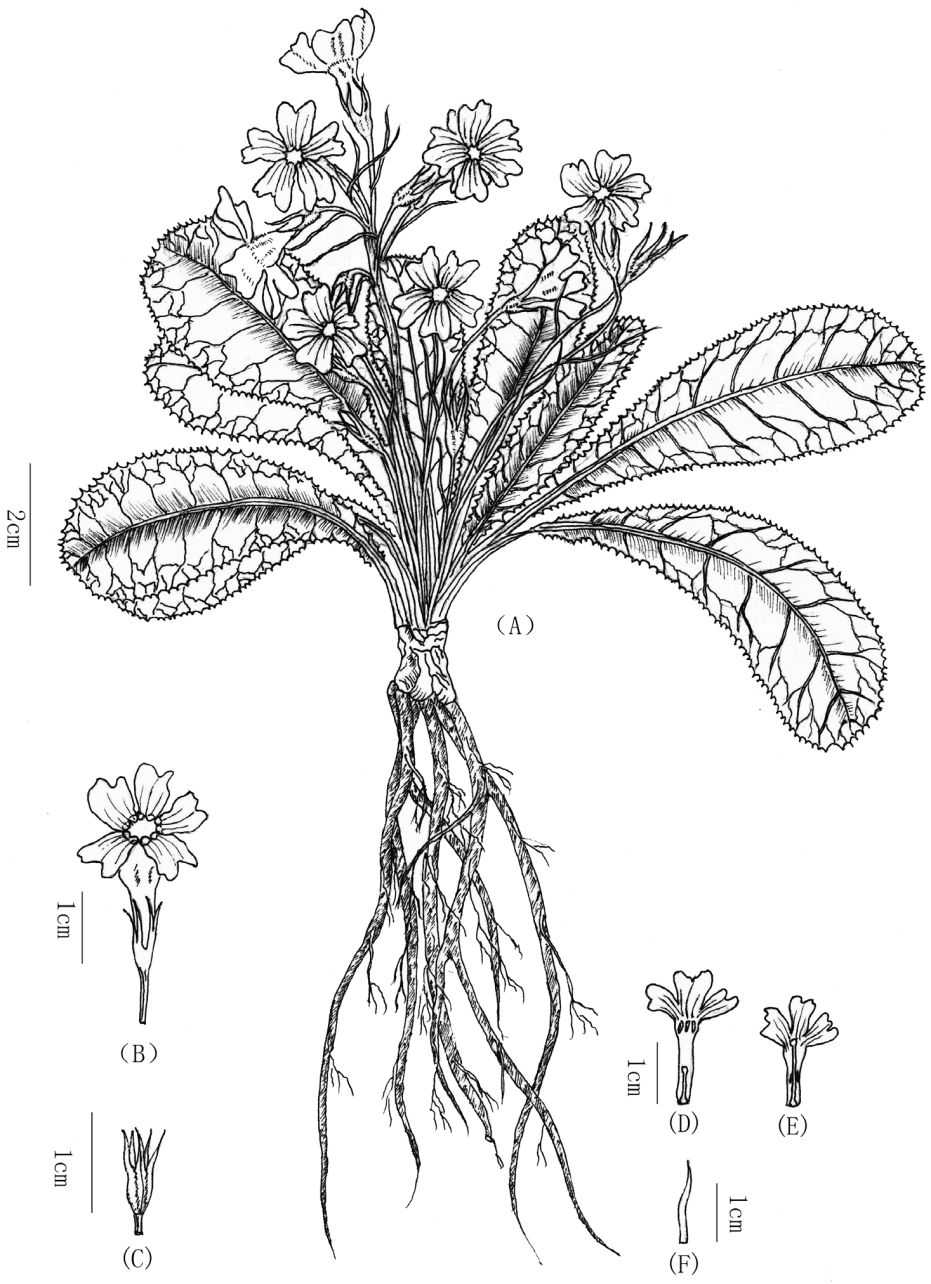
*Primuladongchuanensis* sp. nov. **A** Habit **B** Calyx and corolla **C** Calyx **D** Thrum flower **E** Pin flower **F** Bract.

#### Phenology.

Flowering occurs from late April to early June; fruiting from July to August.

#### Distribution and ecology.

**Figure 2.**
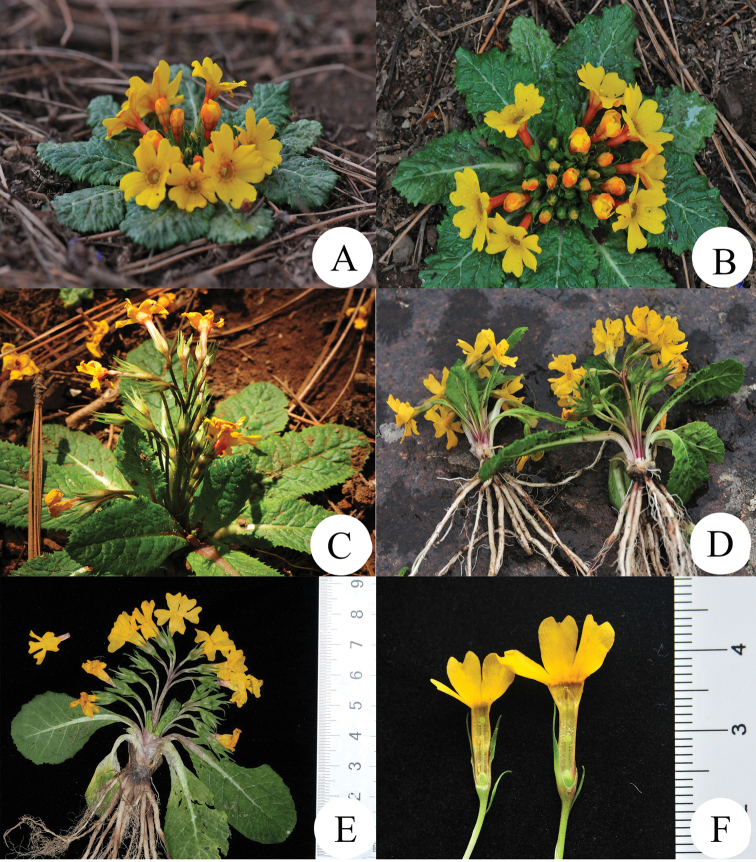
*Primuladongchuanensis* sp. nov. **A, B** Habit in early flowering **C, D** Habit in late flowering **E** specimen of late flowering **F** dissected corolla showing the anthers and stigma, pin flower (left) and thrum flower (right).

*P.dongchuanensis* is only known from the type locality in northern Yunnan, China. The plant has been found on alpine meadow and forest margin at elevation of ca. 3800–4000 m (Fig. 2), associated with *Sibbaldiapurpurea*var.macropetala (Murav.) T.T.Yu & C.L.Li, *Oxygraphisglacialis* (Fisch. ex DC) Bunge, *Androsacerigida* Hand.-Mazz. and *Primulafaberi* Oliv.

#### Etymology.

The epithet of the new species is derived from the name of Dongchuan in northern Yunnan, where the new species was discovered and collected.

#### Vernacular name.

Chinese mandarin: dong chuan bao chun (东川报春)

#### Molecular evidence.

The phylogenetic tree obtained from ML analysis is shown in Figure 4. Phylogenetic analysis showed that the new species clustered with other sampled species of sect. Proliferae and together formed monophyletic clade with a strong support (UFBoot value = 100%, SHaLRT value = 100%), which indicates it is a member of sect. Proliferae, and the tree shows that *P.dongchuanensis* is well differentiated from its close relatives; this is consistent with its special morphological characters in sect. Proliferae.

#### Conservation status.

Currently, *P.dongchuanensis* is only known from the top of Jiaozi Snow Mountain in a single population with fewer than 1000 individuals on ca. 2000 m^2^ occupancy along the alpine meadow. Although there is no obvious population change observed, the original habitat suffered severely from over-grazing based on three field expeditions conducted in 2011, 2016 and 2019. Living collections introduced to Lijiang alpine botanical garden in 2011 were able to flower and set seeds in the following two years, but no individuals were flowering after the fourth year. Other ex-situ conservation actions, such as seed banking, may apply to secure conservation of this unique *Primula* species. According to the guideline of IUCN red list criteria (IUCN 2017), this new species is assessed as ‘Vulnerable’ (VU D1).

## Discussion

**Figure 3.**
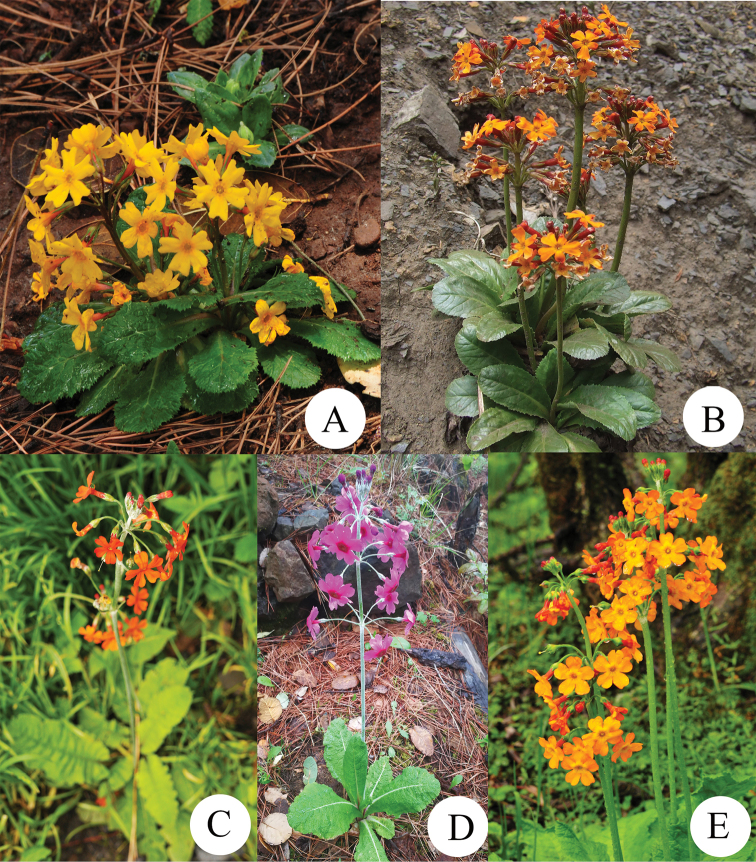
*P.dongchuanensis* and four of its close species **A***P.dongchuanensis***B***P.aurantiaca***C***P.cockburniana***D***P.chungensis***E***P.pulverulenta*.

Sect. Proliferae Pax is a taxonomically well-known group in *Primula*, characterized by numerous whorls of flowers resembling candelabra (Fig. 3) (Chen and Hu 1990). Phylogenetic analyses by using DNA barcoding confirmed the monophyly of sect. Proliferae which could be used in narrowing the scope of identification in *Primula* (Yan et al. 2015). Preliminary molecular phylogenetic analyses in this study supports the view that *P.dongchuanensis* is a member of sect. Proliferae, but its molecular closed relatives are not clear yet. Further research is required to clarify the phylogenetic relationship by using enhanced molecular markers with a wider sampling in this section.

Morphologically *P.dongchuanensis* has unique inflorescences architecture compared to other members of sect. Proliferae. The racemose inflorescence appears in some Primulaspecies, but no report in thesect.Proliferae till the addition of *P.dongchuanensis*, which extended the delimitation of sect. Proliferae and increased our knowledge of the *Primula* diversity in China. Compared to other species of the sect. Proliferae with bigger and upright inflorescences when anthesis begins, *P.dongchuanensis* keeps the racemes in a condensed and short form. This could flow from adaptation to the harsh habit of the mountain top where it is usually very windy and insufficient water in late April and May when it starts anthesis, and the other species of sect. Proliferae are usually found in the open and wet alpine meadow and have a late bloom time.

**Figure 4.**
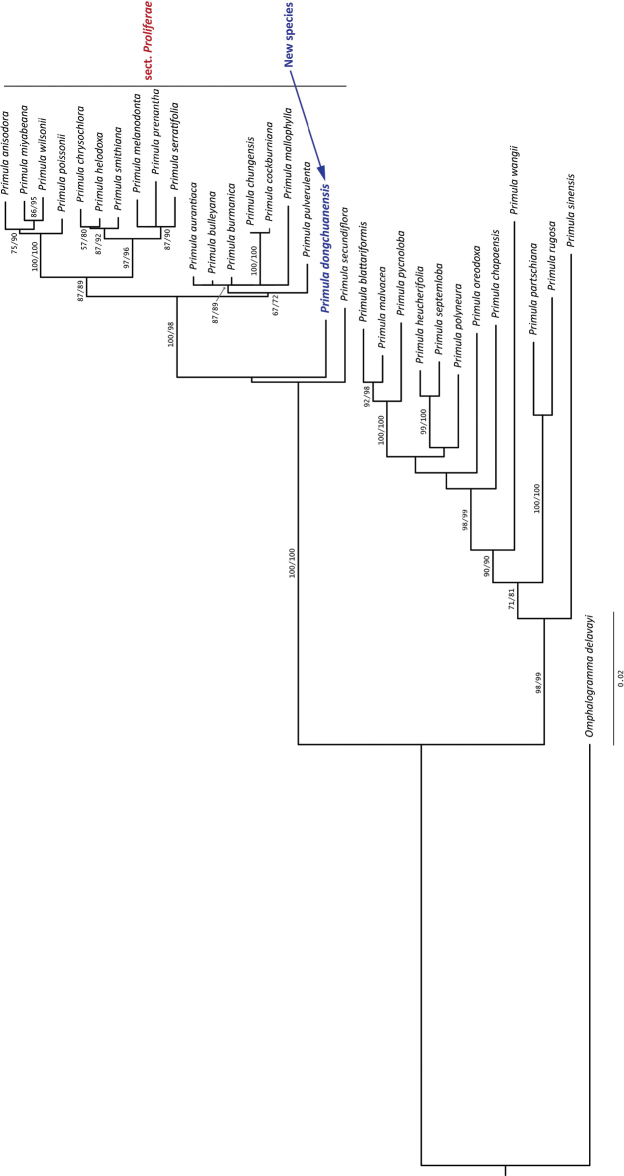
Maximum likelihood tree of new *Primula* species and other *Primula* species based on nuclear ITS, chloroplast *matK* and *trnH-psbA* combined sequenced data, constructed by IQ-TREE under the GTR+G model, clade supports were reported as Shimodaira-Hasegawa approximate Likelihood Ratio Test (SH-alRT)/Ultrafast Bootstrap Approximation (UFBoot), each estimated by 10000 replicates, and only support value more than 50% were reported.

## Supplementary Material

XML Treatment for Primula dongchuanensis

## Appendix 1

**Table:** Information of samples used for phylogenetic inference in this study.

Species	Section	GenBank accession No.			Voucher details
		*matK*	*trnH-psbA*	ITS	
*Primulasinensis* Sabine ex Lindl.	Section Auganthus	JF955776	JN046584	JF978054	Y2010045
*Primulachapaensis* Gapnep.	Section Carolinella	JF955683	JN046494	JF977966	GBOWS681
*Primulapartschiana* Pax	Section Carolinella	JF955748	JN046558	JF978028	Zeng Q.W. s.n.
*Primularugosa* Balakr.	Section Carolinella	JF955764	JN046574	JF978044	Hao 662
*Primulawangii* Chen et C. M. Hu	Section Carolinella	JF955791	JN046599	JF978067	Hao 666
*Primulaheucherifolia* Franch.	Section Cortusoides	JF955715	JN046526	JF977995	Y2010035
*Primulapolyneura* Franch.	Section Cortusoides	JF955749	JN046559	JF978029	GLM103026
*Primulaseptemloba* Franch.	Section Cortusoides	JF955767	JN046575	JF978045	Hao & Yan 989
*Primulablattariformis* Franch.	Section Malvacea	JF955666	JN046477	JF977949	Zhao Y.J. 029
*Primulamalvacea* Franch.	Section Malvacea	JF955720	JN046530	JF978000	Zhao Y.J. 069
*Primulaoreodoxa* Franch.	Section Obconicolisteri	JF955740	JN046550	JF978020	Hao 710
*Primuladongchuanensis*	Section Proliferae	MN181436	MN181434	MN181435	ZKWu2016060
*Primulaanidosora* Balf. f. et Forr.	Section Proliferae	KP638609	KP638689	KP638569	Y2013062
*Primulaaurantiaca* W. W. Smith et Fletcher	Section Proliferae	HM018224	HM018469	HM018175	Hao 536
*Primulabulleyana* Forr.	Section Proliferae	HM018235	HM018480	HM018186	Wu Z.K. 2004018
*Primulaburmanica* Balf. f. et Word	Section Proliferae	KP638614	KP638694	KP638574	Y2013010
*Primulachrysochlora* Balf. f. et Word	Section Proliferae	KP638616	KP638696	KP638576	Y2011005
*Primulachungensis* Balf. f. et Word	Section Proliferae	HM018226	HM018471	HM018177	Hao 465
*Primulacockburniana* Hemsl.	Section Proliferae	KP638621	KP638701	KP638581	Hao 1037
*Primulahelodoxa* Balf. f.	Section Proliferae	HM018228	HM018473	HM018179	Wu Z.K. 2005034
*Primulamallophylla* Balf. f.	Section Proliferae	KP638624	KP638704	KP638584	Y2011084
*Primulamelanodonta* W. W. Smith	Section Proliferae	KP638626	KP638706	KP638586	Y2011070
*Primulamiyabeana* Ito et Kawakami	Section Proliferae	HM018222	HM018467	HM018173	H280
*Primulapoissonii* Franch.	Section Proliferae	HM018241	HM018486	HM018192	Wu Z.K. 2004011
*Primulaprenantha* Balf. f. et W. W. Smith	Section Proliferae	KP638632	KP638712	KP638592	GLM092452
*Primulapulverulenta* Duthie	Section Proliferae	HM018219	HM018464	HM018170	Hao 230
*Primulasecundiflora* Franch.	Section Proliferae	HM018254	HM018499	HM018205	Ge & Yuan 2003T-5
*Primulaserratifolia* Franch.	Section Proliferae	HM018221	HM018466	HM018172	Hao 484
*Primulasmithiana* Craib	Section Proliferae	HM018220	HM018465	HM018171	Hao 640
*Primulawilsonii* Dunn	Section Proliferae	KP638643	KP638723	KP638603	Y2013004
*Primulapycnoloba* Bur. et Franch.	Section Pycnoloba	JF955759	JN046569	JF978039	Hao 766
*Omphalogrammadelavayi* (Franch.) Franch.		KP638606	KP638686	KP638566	Y2013044
